# The Clinicopathological and Survival Profiles Comparison Across Primary Sites in Acral Melanoma

**DOI:** 10.1245/s10434-020-08418-5

**Published:** 2020-04-06

**Authors:** Xiaoting Wei, Di Wu, Hang Li, Rui Zhang, Yu Chen, Hong Yao, Zhihong Chi, Xinan Sheng, Chuanliang Cui, Xue Bai, Zhonghui Qi, Ke Li, Shijie Lan, Lizhu Chen, Rui Guo, Xinyu Yao, Lili Mao, Bin Lian, Yan Kong, Jie Dai, Bixia Tang, Xieqiao Yan, Xuan Wang, Siming Li, Li Zhou, Charles M. Balch, Lu Si, Jun Guo

**Affiliations:** 1 Key Laboratory of Carcinogenesis and Translational Research (Ministry of Education/Beijing), Department of Renal Cancer and Melanoma, Peking University Cancer Hospital and Institute, Haidian District, Beijing, China; 2 Cancer Center, The First Hospital of Jilin University, Changchun, China; 3 Department of Dermatology, National Clinical Research Center for Skin and Immune Diseases, Peking University First Hospital, Beijing, China; 4 Department of Colorectal Surgery, Liaoning Cancer Hospital and Institute, Cancer Hospital of China Medical University, Shenyang, China; 5 Department of Medical Oncology, Fujian Cancer Hospital and Fujian Medical University Cancer Hospital, Fuzhou, China; 6 Department of Cancer Biotherapy Center, Yunnan Cancer Hospital, The Third Affiliated Hospital of Kunming Medical University, Kunming, China; 7 University of Texas MD Anderson Cancer Center, Houston, TX USA

## Abstract

**Background:**

The clinicopathological and survival profiles across primary sites in acral melanoma (AM) are still controversial and unclear.

**Methods:**

This is a multi-center retrospective study. Clinicopathological data of AM patients diagnosed between 1 January 2000 and 31 December 2017 from 6 large tertiary hospitals in China were extracted. Chi square tests were used to compare basic characteristics between primary sites of sole, palm and nail bed. Melanoma-specific survival (MSS) differences based on primary sites were compared by log-rank tests and multivariate Cox regressions were used to identify prognostic factors for MSS.

**Results:**

In total, 1157 AM patients were included. The sole group had a more advanced initial stage, deeper Breslow thickness, higher recurrence rate and distant metastases risk (all *P* < 0.05). The proportion of age < 65 years and ulceration were statistically lower in nail bed and palm groups, respectively. A total of 294 patients underwent sentinel lymph node biopsy and rates of positive SLN status had no statistical difference across primary sites. Among 701 patients with genetic profiles, the mutational frequency of BRAF, C-KIT, and PDGFRA were similar except for NRAS (higher in sole group, *P* = 0.0102). The median MSS of sole, nail bed and palm patients were 65.0 months, 112.0 months, and not reached, respectively (log-rank *P* = 0.0053). In multivariate analyses, primary site, initial stage, ulceration and recurrence were the prognostic factors for MSS in overall population, but the statistical significance varied over primary sites.

**Conclusions:**

Substantial clinicopathological and survival heterogeneities exist across different primary sites in the AM population. Sole melanoma has worse prognosis compared with palm and nail bed subtypes.

Melanoma is a malignancy with a steadily increasing incidence worldwide. Acral melanoma (AM) is a term referring to the melanoma affecting soles, palms, and nail beds.^1^ It is documented as the least common subtype in Caucasians but constitutes up to 50–75% in populations of color.^2^^–^5

Compared with melanoma from other cutaneous sites, AM is associated with a worse survival outcome owing to late detection and negative clinical features.^3^,^6^^–^8 Characteristics of AM have been depicted in many recent studies,^3^,^5^,^6^,^8^,^9^ e.g., deeper Breslow thickness, more frequent ulceration, and more advanced clinical stage at diagnosis.^3^,^8^,^9^

Although acral melanoma is a distinctive subtype, notable variations exist within this population. Previous studies focusing on clinical, genetic and survival discrepancy across different primary sites in AM have yielded controversial results. Nunes et al.^10^ analyzed 529 Brazilian AM patients and reported that the clinical profiles including age, sex, stage, Breslow depth and ulceration status were not significantly different between volar and subungual melanoma. On the other hand, an analysis of 177 AM patients in Korea^11^ showed that the ulceration proportion in the subungual group was higher. Haugh et al.^12^ detected the gene mutational profile in AM patients and found that nail bed melanoma was more likely to have CDK4 copy number aberration and lower frequency of BRAF mutation than melanoma originating on palms. Another report^13^ showed that the prevalence of BRAF mutation between palm and other subgroups in AM was similar but the NRAS mutation frequency was significantly higher in palm melanoma. As for the survival differences, Zebary et al.^14^ believed that sole melanoma had worse survival compared with other subtypes based on a sample of Swedish AM patients. However, tumor location was not considered a prognostic factor for survival in another two reports.^2^,^15^ On the whole, no agreement has been reached on the clinical and survival profiles between different primary anatomical lesions.^10^,^16^

In this study, our objective was to elucidate and summarize the clinicopathological and survival features across primary sites in AM patients based on a Chinese population.

## Patients and Methods

### Patients

This is a multi-center retrospective study incorporating 6 large tertiary hospitals in China. Patients diagnosed with stage I–IV or TxN0M0 AM between 1 January 2000 and 31 December 2017 were enrolled with the following clinical data collected: age, sex, initial disease stage [Based on American Joint Committee on Cancer (AJCC) 8th cutaneous melanoma staging system, stage I–IV. Specifically, to ensure accuracy, pathological stage was used if the patient underwent sentinel lymph nodes biopsy (SLNB); otherwise clinical stage), ulceration status, Breslow thickness, primary site, SLNB and SLN status, locoregional recurrence, in-transit metastasis, distant metastatic sites, gene mutation type, and treatment regimen(s). Patients without complete demographic characteristics data and/or certain primary site specification were excluded. The study was approved by the Institutional Review Board of the Peking University Cancer Hospital & Institute.

### Statistical Analyses

Basic characteristics across different primary sites were compared by Chi square tests or Fisher’s exact tests as appropriate. Kaplan–Meier methods and log-rank tests were used for the comparison of melanoma-specific survival (MSS) between different groups. Unless otherwise specified, log-rank tests were stratified by stage due to consideration of the confounding effect. MSS was defined as the time from initial diagnosis date to the melanoma-specified death date. If the patient was lost to follow-up or still alive then survival was censored at the last date that the patient was known to be alive. MSS was censored at the death date if the patient died from reasons other than melanoma. As SLNB surgical techniques have evolved and matured in recent years, we also performed the log-rank test (stratified by SLNB status, positive vs negative) in patients undergoing SLNB to validate the comparison results.

A multivariate Cox proportional hazard model was used to explore the following prognostic factors: primary site (soles vs palms vs nail beds), age category (< 65 years vs ≥ 65 years), sex (male vs female), ulceration status (present vs absent), stage (stage I vs II vs III vs IV vs TxN0M0), Breslow thickness classification (T1 vs T2 vs T3 vs T4), recurrence (yes vs no). Hazard ratios (HRs) and 95% confidence interval (95% CI) were provided. Since this study covered a large time period and change of treatment modalities should be considered, we generated a dummy variable regarding systemic treatment (if the patient had received immunotherapy or targeted therapy then 1; else 0) as a covariate to control potential confounding effect in the Cox regressions. Meanwhile, we also performed the Cox regression in the subgroup undergoing SLNB (initial stage I–III) with the covariates of primary site, age category, sex, ulceration status, recurrence, Breslow thickness classification, and SLN status.

All analyses were performed using Statistical Analysis System (SAS) software version 9.4 (SAS Institute, Inc., Cary, NC), and a two-tailed *P* value < 0.05 was considered statistically significant.

## Results

### Patient Characteristics

A total of 1157 AM patients were enrolled for this study. Basic characteristics are summarized in Table 1. The number of patients in sole, palm and nail bed groups were 792 (68.5%), 95 (8.2%) and 270 (23.3%), respectively. Overall median age was 56 years old, and 57, 55 and 54 years old for patients with tumor sites of sole, palm and nail bed, respectively. The nail bed population had a higher proportion of age < 65 years old (*P* = 0.0142). Distributions of sex profile was similar irrespective of tumor site. At initial diagnosis, sole patients had a more advanced stage (stage III/IV constituted 33.2% for soles, 16.8% for palms and 24.1% for nail beds. Chi square test *P* = 0.0003) and higher proportion of ulceration (*P* = 0.0248). The overall mean/median of Breslow thickness was 4.2/3.0 mm, and the thickness was the deepest in the sole population (*P* = 0.0067). About 1/4 patients underwent SLNB in populations with the primary sites of sole or nail bed, higher than that of palm group (*P* < 0.0001). The rate of positive SLN status in patients receiving SLNB was not statistically different among these 3 groups.

**Table 1:** Basic characteristics

Indicators	Soles *N* (%)	Palms *N* (%)	Nail beds *N* (%)	Chi square *P* value
Age				
Mean/median (25th–75th percentile)	56.2/57 (48–65)	55.3/55 (50–65)	52.8/54 (44–62)	
< 65 years	581 (73.4)	68 (71.6)	221 (81.9)	0.0142
≥ 65 years	211 (26.6)	27 (28.4)	49 (18.1)	
Sex				0.4434
Male	433 (54.7)	55 (57.9)	138 (51.1)	
Female	359 (45.3)	40 (42.1)	132 (48.9)	
Initial stage				0.0004
TxN0M0	69 (8.7)	4 (4.2)	38 (14.1)	
Stage I	127 (16.0)	22 (23.2)	52 (19.3)	
Stage II	333 (42.0)	53 (55.8)	115 (42.6)	
Stage III	211 (26.6)	14 (14.7)	49 (18.1)	
Stage IV	52 (6.6)	2 (2.1)	16 (5.9)	
Ulceration status				0.0248
Absent	317 (40.0)	37 (38.9)	98 (36.3)	
Present	475 (60.0)	56 (58.9)	171 (63.3)	
Missing	0	2 (2.1)	1 (0.4)	
Breslow thickness				
Mean/median (25th–75th percentile)	4.3/3.0 (2.0–5.0)	3.9/3.7 (2.0–5.0)	4.0/3.0 (1.4–5.5)	
≤ 1 mm (T1)	88 (13.2)	14 (16.3)	41 (19.8)	0.0067
> 1–2 mm (T2)	125 (18.7)	14 (16.3)	38 (18.4)	
> 2–4 mm (T3)	203 (30.4)	22 (25.6)	47 (22.7)	
> 4 mm (T4)	252 (37.7)	36 (41.9)	81 (39.1)	
SLNB				
Yes	211 (26.6)	17 (17.9)	66 (24.4)	< 0.0001
No	581 (73.4)	66 (69.5)	204 (75.6)	
Missing	0	12 (12.6)	0	
SLN status^a^				0.5746
Negative	149 (70.6)	14 (82.4)	48 (72.7)	
Positive	62 (29.4)	3 (17.6)	18 (27.3)	
Recurrence				
Yes	276 (34.8)	18 (18.9)	68 (25.2)	< 0.0001
No	516 (65.2)	44 (46.3)	202 (74.8)	
Missing	0	33 (34.7)	0	
In-transit metastasis				
Yes	29 (3.7)	2 (2.1)	5 (1.9)	0.5742
No	760 (96.0)	93 (97.9)	264 (97.8)	
Missing	3 (0.4)	0	1 (0.4)	
Distant metastases^b^				
At least one site	413	24	111	< 0.0001
Lung	209 (50.6)	14 (58.3)	62 (56.4)	0.4619
Liver	74 (17.9)	4 (16.7)	22 (20.0)	0.8625
Brain	23 (5.6)	1 (4.2)	5 (4.5)	0.8844
Bone	68 (8.6)	3 (3.2)	15 (5.6)	0.0660
Non-regional lymph nodes	215 (52.1)	11 (45.8)	48 (43.6)	0.2663
Other sites	39 (9.4)	6 (25.0)	13 (11.8)	0.0496
Gene mutation^c^				
BRAF	71 (14.6)	7 (9.3)	11 (7.8)	0.0651
C-KIT	43 (8.9)	8 (10.7)	17 (12.1)	0.5068
NRAS	60 (12.4)	1 (1.3)	12 (8.5)	0.0102
PDGFRA	4 (0.8)	0	4 (2.8)	0.0867

The sum of percentage may not equal 100 because of rounding. SLNB: sentinel lymph node biopsy

^a^Only for those patients undergoing SLNB

^b^Include initial and recurrent metastases. The proportions of lung, liver, brain, bone, non-regional lymph nodes and other sites metastases were based on patients with distant metastases

^c^Percentage was calculated only for those patients receiving gene detection

Patients with AM from soles had higher recurrence rates but similar in-transit metastasis risk compared with the other 2 groups (*P* < 0.0001 and *P* = 0.5742). Distant metastases occurred in about 52% of sole patients (including initial and recurrent metastases); significantly higher than the proportion in palm (25.3%) or nail bed populations (41.1%). The lung and non-regional lymph node were the two most common sites of metastases. But the proportions of lung, liver, brain, bone, and non-regional lymphatic metastases were similar across the three groups in those patients having distant metastases.

With respect to gene mutational profile, in total 701 patients underwent gene detection. The frequency of NRAS mutation was higher in the sole group (12.4% vs 1.3% vs 8.5%, *P* = 0.0102). No statistical significance was found in comparison of gene mutational frequency of BRAF, C-KIT, and PDGFRA across the 3 groups.

### Survival Profile

**Fig. 1:**
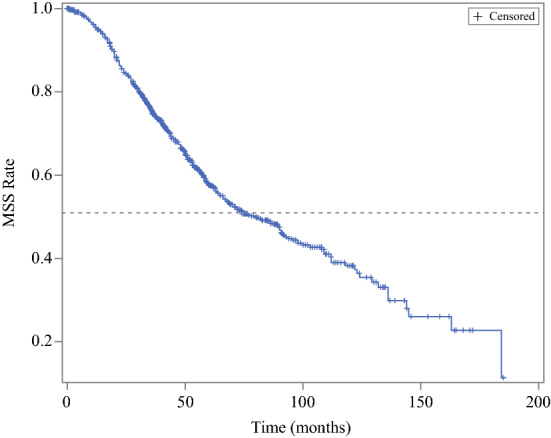
MSS Kaplan–Meier curve of AM patients. The *dashed line* is *y* = 0.5

The Kaplan–Meier curve for melanoma-specific survival for the AM population is shown in Fig. 1. Within the entire cohort, median MSS was 80.1 months (95% CI 68.6, 91.0), and the 1-, 5- and 10-year survival rates were 95.8%, 58.2%, and 38.3%, respectively.

**Fig. 2:**
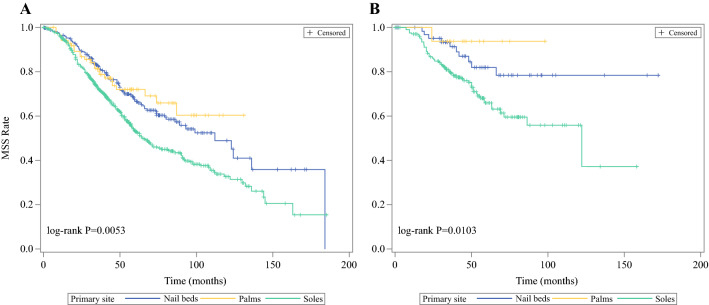
MSS Kaplan–Meier curves of AM patients by primary site. **a** In overall population, stratified by stage. **b** In subpopulation undergoing SLNB, stratified by SLN status (positive vs negative)

Regarding the survival differences across primary sites, the median MSS of patients with the primary site of sole was only 65.0 months (95% CI 59.0, 74.1), significantly shorter than nail bed (112.0 months, 95% CI 86.0, 184.0), and palm (median MSS was not reached) groups (stage-stratified log-rank *P* = 0.0053, Fig. 2a). The 10-year survival rates (95% CI) were 32.8% (27.2%, 38.5%), 60.4% (45.1%, 75.8%), and 48.9% (38.5%, 59.3%) for sole, palm and nail bed groups, respectively. We also performed a log-rank test in the population undergoing SLNB (Fig. 2b), and sole melanoma still had the poorest survival (median MSS: 122.1 months for sole; not reached for palm and nail bed, log-rank *P* = 0.0103).

### Prognostic Factors in AM Population

Potential prognostic factors in the AM population were examined by a Cox proportional hazard model with stepwise method (Table 2). Regression based on the entire cohort showed that primary site, initial stage, ulceration status, and recurrence were the prognostic factors for MSS. But the statistical significance of some factors varied when Cox regressions were performed by primary site. More advanced stages (stages III and IV) had higher mortality risk regardless of primary site. In sole and nail bed populations, patients with tumor ulceration and recurrence of disease had worse prognosis. Although there was no statistical significance in the overall population, an age of ≥ 65 years and male sex predicted worse MSS outcomes in nail bed and sole groups, respectively.

**Table 2:** Cox regression results of prognostic factors for MSS

Groups	Overall	Soles	Palms	Nail beds	Overall_SLNB
	HR (95% CI)	HR (95% CI)	HR (95% CI)	HR (95% CI)	HR (95% CI)
Primary site (soles vs nail beds)	**1.373** (1.105, 1.706)	–	–	–	**2.537** (1.293, 4.976)
Age (≥ 65 vs < 65 years)	1.192 (0.970, 1.464)			**1.812** (1.106, 2.970)	
Sex (male vs female)	1.193 (0.991, 1.436)	**1.259** (1.019, 1.555)			
Stage (II vs I)	**2.073** (1.398, 3.074)				–
Stage (III vs I)	**4.795** (3.240, 7.096)	**2.550** (2.019, 3.222)	**3.213** (1.137, 9.080)	**2.590** (1.598, 4.197)	**–**
Stage (IV vs I)	**11.288** (7.175, 17.758)	**4.961** (3.493, 7.045)	**43.070** (6.605, 280.840)	**25.000** (12.026, 51.973)	**–**
Stage (TxN0M0 vs I)	**2.383** (1.537, 3.694)				–
Ulceration status (present vs absent)	**1.308** (1.066, 1.604)	**1.470** (1.180, 1.832)		**1.970** (1.212, 3.202)	
Recurrence (yes vs no)	**1.659** (1.367, 2.014)	**1.755** (1.416, 2.177)		**2.165** (1.399, 3.350)	**1.804** (1.096, 2.968)
Breslow thickness (T4 vs T1)	**–**	**–**	–	**–**	**1.736** (1.074, 2.804)
SLN status (positive vs negative)	**–**	**–**	–	**–**	**3.677** (2.267, 5.965)

Overall_SLNB represents the subpopulation undergoing SLNB. The factor of recurrence included local and/or regional recurrence. “–” indicates not applicable; *HR* hazard ratio; Cox proportional hazard model with stepwise method, with the entry and stay *P* threshold values of 0.8 and 0.1, respectively; numbers in bold indicate *P* < 0.05

We also carried out a subgroup analysis among the patients who underwent SLNB to identify prognostic factors and to see whether similar factors remained significant (Table 2, last column). Factors of age, sex and ulceration status showed no significance. However, the HR of sole vs nail bed populations increased from 1.373 to 2.537 and was still statistically significant. Patients with recurrence of the disease (HR = 1.804, *P* = 0.0203), Breslow thickness > 4 mm (T4 vs T1, HR = 1.736, *P* = 0.0242) and a positive result for SLN (HR = 3.677, *P* < 0.0001) had worse prognosis.

## Discussion

In this study, we depicted and compared the clinicopathological and survival profiles of patients with acral melanoma from soles, palms, and nail beds. Our detailed analyses of clinical, pathological, and survival data demonstrate substantial heterogeneity across the three primary sites in AM patients. To the best of our knowledge, this study has the largest sample size from the AM population of Asia.

Among 1157 AM patients, 68.5% of patients were reported as sole melanoma at diagnosis in our study. The constitution percentages of different primary sites were consistent with the reports of Haugh et al. and Curtin et al.^12^,^17^ With respect to age, the overall median age of the patients at time of diagnosis was 56 and the sole group had an older median/mean age than the other two groups, which is similar to another study result.^17^ Nunes et al.^10^ analyzed 157 Brazilian AM patients; the age at diagnosis was older than ours and the age distribution between palms and nail beds was similar. This was not consistent with our comparison results. As for the Breslow thickness, median thickness in this present study was 3.0 mm and the proportion of thickness > 2 mm was higher in melanoma on soles and palms than in the nail bed group. The distribution of sex was similar across the three groups, and this was comparable to another study^7^ on 142 Chinese AM patients. Both the proportion of females and ulcerated rate were reported higher in Nunes et al.’s^10^ report; but in our study, the correlation between melanoma arising on different lesions was statistically significant for ulceration but not for sex. Although more patients underwent SLNB in the sole group, the positive percentages of SLN status were not significantly different among the populations with different tumor sites.

With regard to the clinical stage and metastasis features, sole melanoma had a more advanced stage and about 1/3 presented as stage III/IV at diagnosis. The stage III/IV distribution for the rest 2 groups were similar. Another study,^10^ however, showed that the proportion of stage III/IV in the palm group was threefold higher than that in the nail bed group, which may be due to race variation. The overall distant metastases percentage in our study was comparable with previous literature^2^,^18^ but the sole population had a higher proportion of distant metastases. No statistical difference was found for metastases of lung, liver, brain, bone, or non-regional lymph nodes across the 3 acral subgroups.

AM has been regarded as a worse and more aggressive subtype due to delay of diagnosis, socioeconomic factors, negative prognostic, and genetic features.^3^,^6^,^7^ When it comes to the MSS profile differences among primary lesions, median MSS of sole patients was 65.0 months, while it was 112.0 months for nail bed patients and not reached for the palm group. It is evident that the survival of the sole population was worse than that of palm and nail bed AM populations. The comparison results were further validated and confirmed in the population undergoing SLNB. In Cox proportional hazard regression, primary site was also an independent prognosis factor for MSS. Zebary et al.^14^ analyzed 88 Swedish AM patients and observed that the survival of those with sole primary sites was worse as well. Based on our findings, the distribution of clinical characteristics can in part explain the survival difference. First, the sole group had higher recurrence and distant metastases risk compared with the populations with palm and nail bed melanoma. Second, sole patients had deeper Breslow thickness and a more advanced stage when diagnosed with melanoma. These are in keeping with Bradford et al.’s^3^ and another 3 reports.^11^,^18^,^19^ Meanwhile, previous studies^11^,^20^,^21^ reported that more physical stress and trauma occurred in patients with sole primary sites, leading to higher recurrence and mortality risk. Inconsistent with our results, however, was the report by Lv et al.;^7^ they assessed 142 Chinese AM patients and found that sites of lesion had no significant impact on overall survival (OS). But this result was unpersuasive because of the limitation of sample size.

Multiple publications^2^,^3^,^5^,^10^,^14^,^22^,^23^ have reported that age, level of invasion, ulceration status, recurrence, stage, etc. were the prognostic factors for AM survival, but the statistical significance of some variables were conflicting among those studies. For example, level of invasion was not an independent prognostic factor in Teramoto et al.’s study,^2^ but the finding was opposite in another report.^14^ Our site-based Cox regressions showed that the prognosis of AM varied depending on the primary tumor locations. Stage was the only common prognostic factor across all these 3 groups, in alignment with previous reports.^3^,^5^ On the other hand, and different from the AJCC 8th manual,^24^ both our findings and several other publications^2^,^5^,^10^ showed that the survival trends among less-advanced-stage (stage I, stage II) AM patients were similar. For example, one analysis^5^ of 715 cases identified the 5-year OS rate as 53.3% and 52.7% for AM patients with stage I and stage II, respectively. Based on these results, it is suggested that the applicability of the current AJCC staging system be further verified in the AM population, especially with a larger sample size. Regarding other prognostic factors, tumor ulceration and recurrence indicated worse survival outcomes for sole and nail bed groups, but not for palms. In the sole population, males and females exhibited similar prognoses, but this did not align with the results of Lino-Silva et al.^5^ Higher mortality risk was presented among elder patients in the AM population with nail bed primary sites, but survival was not affected by age in sole and palm melanomas. Regarding the subgroup who underwent SLNB, we found that a positive status of SLN significantly predicted a worse MSS outcome, which is consistent with previous reports on AM and cutaneous melanoma.^10^,^25^,^26^

There are some limitations and strengths in our study. First, only a small proportion of AM patients underwent SLNB in this retrospective study. Although the comparison results were confirmed in this subpopulation, we still should interpret the results cautiously. Second, when Cox regressions among the palm subgroup were performed, the number of MSS events was limited, thus leading to a decrease in statistical power (such as large confidence interval and insignificant *P* value for some factors). On the other hand, this study has the largest sample size from the Asian AM population and we believe that the results can still well represent the current AM profile in Asia.

In conclusion, substantial heterogeneities exist across primary sites in the AM population. Sole group patients had a worse prognosis compared with those with primary sites arising on palms and nail beds. Clinicians should attach importance to these differences when they provide associated treatments. More aggressive regimens should be considered for AM patients with the primary site of soles.
